# Sleep spindles and slow oscillations predict cognition and biomarkers of neurodegeneration in mild to moderate Alzheimer's disease

**DOI:** 10.1002/alz.14424

**Published:** 2025-01-29

**Authors:** Arsenio Páez, Sam O. Gillman, Shahla Bakian Dogaheh, Anna Carnes, Faride Dakterzada, Ferran Barbé, Thien Thanh Dang‐Vu, Gerard Piñol Ripoll

**Affiliations:** ^1^ Sleep, Cognition and Neuroimaging Laboratory Concordia University Montreal Canada; ^2^ Centre de Recherche de l'Institut Universitaire de Gériatrie de Montréal (CRIUGM) Montréal (Québec) Canada; ^3^ Nuffield Department for Primary Care Health Sciences University of Oxford Oxford UK; ^4^ Unitat de Trastorns Cognitius Cognition and Behavior Study Group Hospital Universitari Santa Maria Universitat de Lleida Lleida Spain; ^5^ Translational Research in Respiratory Medicine (TRRM) Hospital Universitari Arnau de Vilanova‐Santa Maria Biomedical Research Institute of Lleida (IRBLleida) Lleida Spain; ^6^ Alzheimer's Disease and Other Cognitive Disorders Unit Neurology Service Hospital Clínic de Barcelona Fundació de Recerca Clínic ‐ Institut d'Investigacions Biomèdiques August Pi i Sunyer (IDIBAPS) Barcelona Spain

**Keywords:** Alzheimer's disease, amyloid beta, biomarkers, cognition, cognitive decline, sleep, sleep spindles, slow oscillations, tau

## Abstract

**INTRODUCTION:**

Changes in sleep physiology can predate cognitive symptoms by decades in persons with Alzheimer's disease (AD), but it remains unclear which sleep characteristics predict cognitive and neurodegenerative changes after AD onset.

**METHODS:**

Using data from a prospective cohort of mild to moderate AD (*n* = 60), we analyzed non‐rapid eye movement sleep spindles and slow oscillations (SOs) at baseline and their associations with baseline amyloid beta (Aβ) and tau and with cognition from baseline to 3‐year follow‐up.

**RESULTS:**

Higher spindle and SO activity predicted significant changes in Aβ and tau at baseline, lower Alzheimer's Disease Assessment Scale Cognitive Subscale (better cognitive performance) score, and higher Mini‐Mental State Examination score from baseline to 36 months. Spindles and SOs mediated the effect of phosphorylated tau 181 (pTau181)/Aβ42 on cognition, while pTau181/aβ42 moderated the effect of spindles and SOs on cognition.

**DISCUSSION:**

Our findings demonstrate that spindle and SO activity during sleep constitute predictive and non‐invasive biomarkers of neurodegeneration and cognition in AD patients.

**Highlights:**

- Sleep spindles predict long‐term cognitive performance in AD.
- Sleep spindle and SOs can be predictive, non‐invasive biomarkers for AD.
- Sleep may be one of the most important modifiable risk factors for AD progression.
- Sleep microarchitecture is a novel therapeutic target for preserving brain heath.
- Sleep physiology can provide novel therapeutic targets to slow AD progression.

## 1 BACKGROUND

Poor sleep has been linked to increased risk of Alzheimer's disease and related dementias (AD/ADRD) and can predict or accelerate the progression of cognitive decline in persons with Alzheimer's disease (AD).^1^, ^2^, ^3^ Up to 66% of persons with AD/ADRD experience poor sleep.^1^, ^2^, ^3^, ^4^ Sleep disorders such as insomnia and sleep apnea often appear in the preclinical stage of AD.^1^, ^2^, ^3^, ^4^ AD patients also experience greater sleep latency (time to fall asleep once in bed), wake time after sleep onset, and number of nighttime awakenings than healthy older adults, along with significant reductions in total sleep time (TST), sleep efficiency, slow‐wave sleep (SWS), and rapid eye movement (REM) sleep.^5^

There is a bidirectional relationship between sleep and AD/ADRD.^6^, ^7^ Sleep plays important roles in the clearance of amyloid beta (Aβ) and tau linked to AD/ADRD.^1^, ^8^ Sleep disturbances have been linked to increased Aβ and tau accumulation in the brain, which can precede the first cognitive symptoms of AD by 15 to 20 years.^1^, ^6^, ^9^ Conversely, Aβ and tau pathology in cortical regions, the hypothalamus, and nuclei regulating sleep‐wake can lead to sleep disturbances and impaired slow‐wave activity (SWA) during non‐rapid eye movement (NREM) sleep, which may also contribute to hippocampus‐dependent cognitive decline in older adults.^6^, ^10^, ^11^

Sleep spindles (SPs) and slow oscillations (SOs) during NREM sleep are also altered in AD and have been linked to AD progression.^12^, ^13^ Sleep SPs are 9 to 16 Hz waxing‐and‐waning oscillations generated within the thalamocortical network and consistently associated with sleep‐dependent memory consolidation, cognition, and sleep continuity.^14^ SP density, duration, and amplitude decrease with age but are further reduced in persons with AD/ADRD.^5^, ^15^, ^16^ Large‐amplitude, low‐frequency slow waves (>75 microV, 0.5 to 4  Hz) and SOs (<1 Hz), largely arising from the prefrontal neocortex, also underlie memory consolidation during sleep.^17^ SOs decrease in number and amplitude in middle‐aged and older adults.^12^, ^18^ These decreases are more pronounced in persons with cognitive decline or AD/ADRD.^12^, ^18^

### 1.1 Evidence gaps and opportunities

Changes in sleep physiology can precede the onset of cognitive changes by years or decades in persons with AD.^4^, ^6^, ^13^, ^19^, ^20^ Sleep assessments, including features of NREM sleep correlated with AD pathology or cognitive decline, offer the potential for early, non‐invasive screening for AD risk before the onset of clinical symptoms and provide novel therapeutic targets to slow AD progression.^7^, ^21^ Plasma, cerebrospinal fluid (CSF), and positron emission tomography (PET) can provide useful biomarkers of Aβ and tau levels in the brain. However, access to PET can be limited, requiring expensive, specialized facilities.^22^ Lumbar puncture for CSF is more accessible but is invasive and carries risk of mild adverse reactions.^22^ Ultra‐sensitive blood‐based assays enable increasingly accurate biomarker measures at lower cost but require blood draws and specialized laboratory equipment.^23^

**RESEARCH IN CONTEXT**

1. **Systematic review**: We searched systematically in PubMed and Embase, conducting extensive reviews of papers reporting sleep, cognition, biomarkers, and disease progression in AD in the preparation of this study and manuscript.
2. **Interpretation**: Sleep may be one of the most important modifiable risk factors for cognitive decline and the progression of AD. Our findings demonstrate that NREM spindle and SO activity are predictive and non‐invasive biomarkers of neurodegeneration and cognition in AD, with potential to provide novel therapeutic targets to slow AD progression and preserve brain heath.
3. **Future directions**: Future studies may
  (a) investigate associations between NREM sleep physiology and AD progression in larger and more diverse samples, extending our findings,
  (b) collect data from PET for topographic interpretation of associations between alterations in spindles and SOs and anatomical distributions of tau pathology and neurofibrillary tangle, and
  (c) investigate the effects of longitudinal changes in sleep physiology on brain health and cognition as AD progresses.

Increasingly, research has explored associations between sleep physiology, AD biomarkers, and cognition in healthy older adults.^11^, ^13^, ^24^, ^25^, ^26^, ^27^, ^28^, ^29^ Less is known about predictive associations between spindles, SOs, biomarkers, and cognition in persons with AD.^6^, ^30^ SP intensity and density have been correlated with episodic memory and cognition in limited samples of up to 15 persons with AD.^31^, ^32^ Inverse relationships between 1 and 2 Hz SWA and CSF tau have also been found in preclinical and early AD.^29^ However, associations between SP and SO characteristics, biomarkers of neurodegeneration, and cognitive performance have not been investigated together in AD patients.

In this study, we investigated associations between baseline NREM SP and SO activity in a sample of 60 persons with mild to moderate AD and (1) AD/ADRD biomarkers at baseline and (2) cognitive performance over 3 years.

We hypothesized that NREM sleep SP and SO activity would predict Aβ42 and tau levels at baseline. We also hypothesized that higher SP and SO activity would predict higher cognitive performance (direct relationship) in persons with mild to moderate AD. Sleep SP and SO activity may constitute predictive, non‐invasive biomarkers of neurodegeneration and cognition in AD patients and novel therapeutic targets to slow AD progression and preserve brain‐heath.

## 2 METHODS

### 2.1 Study design

To test these hypotheses, we performed secondary analyses of data previously collected (from November 2014 to November 2017) in a prospective study of the influence of obstructive sleep apnea (OSA) on the cognitive evolution of persons with AD (“Role of Hypoxia and Sleep Fragmentation in Alzheimer's Disease,” ClinicalTrials.gov NCT02814045).^2^, ^33^ Participant eligibility, recruitment, and data collection methods have been described extensively.^2^, ^33^ Briefly, a cohort of 104 persons with mild to moderate AD/ADRD were recruited in a prospective, observational study at the Cognitive Disorders Unit of Hospital Universitari Santa Maria (Lleida, Spain). Eligible participants were aged ≥60 years old with AD diagnosed according to the National Institute on Aging and Alzheimer's Association criteria.^34^ Exclusion criteria have been described elsewhere and included the use of investigational drugs, beta blockers, antidepressants, neuroleptics, or hypnotics within 15 days of the overnight polysomnography (PSG).^2^, ^33^ None of the participants were taking anticonvulsants, antipsychotics, anxiolytics, hypnotics, or medications for poor sleep, such as benzodiazepine receptor agonists, that might have influenced sleep microarchitecture. Five participants had previously taken antidepressants, but not for at least 15 days before their PSG.

The study was approved by the Institutional Review Board of Hospital Arnau de Vilanova de Lleida (CE‐1218) and conducted according to the Declaration of Helsinki principles. Data from 60 of the participants (30 females) were available for the analyses described in this paper.

### 2.2 Data collection

**FIGURE 1:**
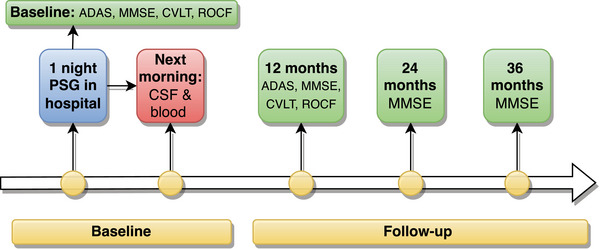
Study flow from baseline to 36 months.

At baseline, participants underwent one‐night PSG (Figure 1). The following morning CSF was collected for biomarkers associated with amyloid deposition, tau pathology, neurodegeneration, axonal damage, synaptic integrity, neuroinflammation, and oxidative damage. At baseline and 12‐month follow‐up visits, participants underwent a battery of functional and neuropsychological assessments, including the Alzheimer's Disease Assessment Scale–Cognitive Subscale (ADAS‐Cog),^35^ California Verbal Learning Test (CVLT), Mini‐Mental State Examination (MMSE),^36^ Rey–Osterrieth Complex Figure (ROCF) test, and others (eg, Cornell Scale for Depression in Dementia^37^ and Neuropsychiatric Inventory [NPI]^38^) capturing mental and physical function.^2^, ^33^ At the 24‐ and 36‐month follow‐up appointments, only the MMSE was administered.

The ADAS‐Cog assesses cognitive and behavioral domains most affected by AD and is a gold standard for assessing the efficacy of treatments targeting dementia.^35^ The MMSE assesses cognitive function and impairment in older adults.^36^ The ROCF test^39^ and CVLT^40^ capture visual and verbal (respectively) learning and memory.

#### 2.2.1 Sleep

PSG was undertaken in Lleida, Spain, using Philips Respironics Alice 6 LDx (34‐channel EEG referenced to the mastoids, 512 Hz sampling rate). Electroencephalography (EEG) records were visually scored by experienced sleep technicians using Phillips G3 Dreamware software (bandpass filter 0.3 to 93.6  Hz, sampling frequency 512 Hz) in 30‐s epochs in one of four stages of sleep (NREM stages 1, 2, or 3 [NREM1, NREM2, NREM3], and rapid eye movement [REM]) following American Academy of Sleep Medicine (AASM) criteria.^41^

#### 2.2.2 Biomarkers

The collection of CSF samples for biomarker analysis has been described previously.^33^ Briefly, CSF samples were collected from participants in the morning, between 8:00 and 10:00 a.m., to avoid variations related to circadian rhythms. Samples were collected in polypropylene tubes, centrifuged at 2000 × *g* for 10 min at 4°C, immediately frozen, and stored within 4 h in a −80°C freezer. The principal AD biomarkers, CSF Aβ42, total tau (t‐tau), and phosphorylated tau 181 (pTau181), were measured using commercial kits (Innotest β‐Amyloid1‐42; Innotest hTAU Ag; and Innotest Phospho‐TAU181P, Fujirebio‐Europe, Ghent, Belgium). All measurements were performed in one round using one batch of reagents by board‐certified laboratory technicians blinded to clinical data. Intra‐assay coefficients of variation were lower than 10% for internal quality control samples (two per plate).

### 2.3 Data analyses

#### 2.3.1 Cognition

For this study, the results of the CVLT, ROCF test, ADAS‐cog, and MMSE were analyzed to best capture the cognitive status and progression of cognitive decline in persons with mild to moderate AD. The ADAS‐cog, CVLT, and ROCF test were available for baseline and 12‐month follow‐up. The MMSE was available for the baseline and 12‐, 24‐, and 36‐month follow‐ups.

#### 2.3.2 Polysomnography and EEG analysis

For the present study, participants’ sleep EEG records were processed using Wonambi version 7.11, an open‐source Python software package developed at the Sleep, Cognition, and Neuroimaging Laboratory (SCNLab) at Concordia University, Montreal (https://github.com/wonambi‐python/wonambi). Participants’ previously scored EEGs were visually checked again by experienced sleep scorers (AP, SG, SB, OW) at the SCNLab, following AASM criteria.^41^

We identified sleep cycles as those periods of sleep containing at least 15 min of NREM sleep and 5 min of REM sleep, except for the first sleep cycle, which could contain at least 1 min of REM sleep.^42^ We then reviewed the EEG sleep recordings for each participant in 30‐s epochs to identify and tag microarousals, artifacts, or instances of poor EEG signal for exclusion from subsequent analyses. Signal aberrations we targeted for removal included poor or dysfunctional signal lasting more than one 1 s (eg, signal popping and flat lining),^41^ excessive muscle artifact or movement, micro‐arousal activity (sudden transient cortical activations during sleep and abrupt shift in EEG frequency lasting longer than 3 s^41^), or periods in an epoch that contained a shift to N1 or wakefulness.

Automatic SO and sleep SP event detection on EEG were performed using another in‐house software package developed at the SCNLab and run on Spyder (version 5.3.3), an open‐source environment in Python. The in‐house software directly incorporates functions from validated sleep SP and slow‐wave detection methods. Based on our review of the literature for SP and SWA in older adults, we undertook sleep SP detection over two central channels (C3‐A2 and C4‐A1)^15^, ^16^ and SO detection over the two frontal channels (F3‐A3, F4‐A1).^43^

An algorithm developed by Staresina et al. (2015) was used to detect SOs on channels F3 and F4 during N2 and N3.^17^ Artifact‐free EEG signals were filtered between 0.16 and 1.25 Hz (zero‐phase infinite impulse response bandpass filter). The duration threshold for potential SOs was 0.8 to 2.0 s for two successive positive‐to‐negative zero crossings, in filtered EEG signals. Following Staresina's criteria, the event amplitude threshold for SOs meeting the duration criteria was amplitudes exceeding the 75th percentile of trough‐to‐peak amplitude between two positive‐to‐negative zero crossings.^17^ We also visually reviewed detected SO events to ensure that they were not false positives.

Sleep SPs were detected during NREM2 and NREM3 (fixed bandwidth 9 to 16  Hz) based on Mölle et al.’s (2011) algorithm.^44^ We chose a 9‐ to 16‐Hz bandwidth based on a review of the literature for SP characteristics in older adults, in mild cognitive impairment (MCI), AD, and other neurodegenerative disorders.^5^, ^45^, ^46^, ^47^, ^48^, ^49^, ^50^, ^51^ These disorders have been associated with greater declines in SP characteristics and physiological changes than are seen with increasing age.^5^, ^31^, ^32^, ^46^, ^47^, ^52^, ^53^, ^54^ We also wanted to capture slow SP activity (9 to 12  Hz)^44^, ^45^, ^55^ in our sample, given evidence of age‐related changes in slow SP activity and their links with cognitive decline.^53^, ^54^ On artifact‐free, filtered EEG signals in channels C3 and C4, the root mean square (RMS) was computed using a 0.2‐s sliding window, then further smoothed using 0.2‐s moving average. Sleep SPs were identified when standard deviations (SDs) of RMS values exceeded 1.5 for 0.5 to 3.0 s. To manually check the accuracy of automatically detected SO and sleep SP events, we visually inspected the identified sleep SPs with Wonambi, which highlighted the previously detected events on filtered EEG signals.

With these methods we extracted data for sleep SP count, density (/30 s), mean duration (s), peak‐to‐peak (PTP) amplitude (µV), power (µV2), and peak power frequency (Hz) and SO density (/30 s), mean duration (s), PTP amplitude (µV), power (µV2), and peak power frequency (Hz) in NREM2 and NREM3. In this paper, we report data for SP duration, density, and power and SO duration, density, and amplitude in NREM2 and NREM3 combined. Data for NREM2 and NREM3 separately are available in Tables S1 to S4.

These characteristics were chosen for their associations with, and ability to predict, cognitive performance.^3^, ^15^, ^16^, ^56^ For example, SP and SO duration and density have been linked to cognitive decline and may be associated with CSF levels of biomarkers for neurodegeneration in healthy older adults.^16^, ^20^, ^49^, ^50^ SP power has been identified as a predictor of cognitive performance in healthy older adults.^57^ Results for the other SP and SO characteristics, such as SP or SO count or peak frequency, are available in Tables S1 to S4.

### 2.4 Statistical analyses

Descriptive statistics, including the mean and SD for parametric data, median and interquartile range (IQR) for non‐parametric data, and differences in these among men, women, and persons with high (<600 ng/L, or AΒ+^58^) or low (>600 ng/L, or AΒ−) Aβ burden at baseline were calculated for participant characteristics, sleep, Aβ42 and tau biomarkers, and cognition‐neuropsychological test scores. The normality of distributions was analyzed using the Shapiro–Wilk test and visual inspection of histograms and kernel density plots, which approximate the probability density of the variable. We expected the biomarker and cognition data to be right or left skewed rather than normally distributed, as may be expected in participants with mild to moderate AD.^59^

#### 2.4.1 Sleep microarchitecture and AD biomarkers

We calculated ratios of CSF pTau181/Aβ42 and total tau/Aβ42 to investigate associations between sleep, AD biomarkers, and cognition.^60^ These CSF biomarker ratios have been associated with increased brain amyloid and show superior agreement with PET amyloid measures.^60^ They have also been shown to be superior to individual biomarkers at predicting the risk and rate of clinical decline and progression to dementia in older adults at risk of AD.^60^

Associations between SO and SP metrics (eg, duration, density, power) and CSF biomarkers (Aβ42, tau [t‐tau, pTau181] and their ratios pTau181/Aβ42 and total tau/Aβ42) at baseline were investigated with generalized linear models (GLMs) with Huber/White/Sandwich robust estimators of variance, reducing the influence of small, non‐zero values that may bias the results of linear regression, increasing robustness against non‐normally distributed error, and accounting for heteroskedasticity in residual distributions.^61^

The association between SO/SP metrics and biomarkers was modeled with one primary biomarker outcome variable (eg, pTau181/Aβ42), sleep SP or SO characteristics, and covariates for age (continuous), sex (binary), and apnea‐hypopnea index (continuous). The apnea‐hypopnea index (AHI) was included as a covariate in all analyses. The sample was drawn from a population of older adults with a high prevalence of OSA, and hypoxia severity has been associated with amyloid deposition and increased risk of AD in persons with OSA.^8^ For every regression model there were at least 10 participants per dependent variable or covariate in the model, avoiding overfitting that may bias regression results.

#### 2.4.2 Sleep microarchitecture and cognition

The associations between SO/SP metrics in NREM2 and NREM3 and cognitive assessments, including the CVLT, ROCF test, ADAS‐Cog, and MMSE were conducted cross‐sectionally (baseline) with GLMs and Huber/White/Sandwich robust estimators of variance. Longitudinal analyses from baseline to 12 months for the CVLT, ROCF test, ADAS‐cog, and baseline to 12, 24, and 36 months for the MMSE were also conducted with GLMs, adjusting for baseline scores. The odds of decline in MMSE scores per unit of change in a SP or SO metric of interest were calculated with the odds ratio of a decline in MMSE from baseline to 36 months.

In all regression models, we checked for multicollinearity among sleep SP and SO characteristics with the variance inflation factor (VIF), maintaining VIF at <2 for all models (no to low correlation).^62^ The normality of regression residuals was assessed with Shapiro–Wilk tests. Only the residuals for pTau181/Aβ42 were non‐parametrically distributed. We visually re‐inspected the data for pTau181/Aβ42 and used the Tukey Ladder of Powers to identify the most appropriate transformation for that outcome variable. We applied a square root transformation to pTau181/Aβ42 and reran the regression model with the transformed variable, yielding normally distributed residuals. We corrected for multiple comparisons in all regression analyses with a Benjamini‐Hochberg false discovery rate (FDR).^63^

Moderation and mediation analyses were undertaken to explore whether there were mediating or moderating roles for biomarkers in the relationship between SP and SO activity and cognitive performance or a moderating or mediating role for SP and SO activity in the relationship between biomarkers and cognitive performance. These exploratory analyses were carried out in STATA using Sobel–Goodman tests of mediation following Preacher and Hayes’ recommendations.^64^ For moderation analyses, we ran GLM regression models with interaction terms. We explored these with the MMSE, ADAS‐Cog, CVLT, and ROCF test at baseline and follow‐up periods and the pTau181/Aβ42 ratio, given its associations with AD progression and superiority at predicting clinical decline and progression to dementia versus individual biomarkers.^60^

## 3 RESULTS

The 60 participants (30 female) in this study had a mean age of 74.7 at baseline. There were no significant differences between males and females in age, body mass index, educational attainment, prevalence of diabetes, depression, or obstructive sleep apnea (Table 1). Participants with Aβ42 < 600 pg/mL at baseline had a higher prevalence of OSA than participants with Aβ4 > 600 pg/mL (Table S5). Participants had a median Aβ42 of 516 pg/mL and no statistically significant differences between males and females in Aβ42 CSF or tau levels or ratios of Aβ42 to tau. No statistically significant differences were found between men and women on the Cornell Scale for Depression in Dementia or NPI.

**TABLE 1:** Participants’ characteristics at baseline.

Sample	Male (*n* = 30)	Female (*n* = 30)	Total (*n* = 60)	*p*
Age	75.5 ± 5.0	74.0 ± 5.0	74.7 ± 5.0	0.26
Body mass index (BMI)	27 (24 to 29)	28 (24‐32)	27 (24 to 32)	0.71
Depression	6 (20.0%)	12 (40.0%)	18 (30%)	0.09
Diabetes	7 (23.3%)	3 (10%)	10 (16.7%)	0.17
**Education** (≥ high school)	5 (16.7%)	5 (16.7%)	10 (16.7%)	1.00
0: No formal education	2 (6.7%)	2 (6.7%)	4 (6.7%)	
1: Primary school	23 (76.7%)	23 (76.7%)	46 (76.7%)	
2: High school	4 (13.3%)	4 (13.3%)	8 (13.3%)	
3: University	1 (3.3%)	1 (3.3%)	2 (3.3%)	
**Smoking history**	11 (36.7%)	2 (6.7%)	41 (68.3%)	0.005
0: Never	19 (63.3%)	28 (93.3%)	47 (78.3%)	
1: Current	2 (6.7%)	0 (0.0%)	2 (3.3%)	
2: Former (>6 months ago)	9 (30.0%)	2 (6.7%)	11(18.3%)	
Obstructive sleep apnea (OSA)	24 (96.0%)	23 (88.5%)	47(78.3%)	0.25
**Apnea hypoxia index** (*n*/h total sleep time [TST])	38.14 ± 23	29.24 ± 22.8	33.7 ± 23.14	0.14
0 to 4.9	1 (3.3%)	2 (6.7%)	3 (5%)	
5 to 14.9	4 (13.3%)	7 (23.3%)	11 (18.33%)	
15 to 30	9 (30.0%)	10 (33.3%)	19 (31.67%)	
≥30	16 (53.3%)	11 (36.7%)	27 (45%)	
**AD drugs**	26 (86.7%)	27(90%)	53 (88.3%)	0.69
None	4 (13.3%)	3 (10%)	7 (11.7%)	
Rivastigmina	9 (30%)	9 (30%)	18 (30%)	
Donepezil	17 (56.7%)	15 (50%)	32 (53.3%)	
Memantine	0	3 (10%)	3 (5.8%)	
**CSF values, biomarkers (pg/mL)**				
Amyloid beta (Aβ42)	506 (417 to 609)	532 (398 to 627)	516 (411 to 618)	0.62
Phosphorylated tau (pTau181)	61 (48 to 98)	90 (54 to 103)	82 (50 to 100)	0.90
Total tau	487.4 ± 287.7	599.2 ± 276.5	543.3 ± 285.0	0.16
PTau181/Aβ42 ratio	0.14 ± 0.07	0.17 ± 0.08	0.16 ± 0.74	0.25
Total tau/Aβ42 ratio pg/mL	0.87 (0.52 to 1.34)	1.10 (0.70 to 1.63)	0.96 (0.55 to 1.48)	0.27
**Cognition**				
ADAS‐cog total score	28 (26 to 32)	29 (25 to 31)	29 (25 to 31)	0.89
MMSE	23.4 ± 2.3	23.0 ± 2.4	23.2 ± 2.4	0.55
**Neuropsychiatric**				
Cornell Scale (CSDD)	7 (2 to 11)	6 (3 to 11)	7 (3 to 11)	0.93
Neuropsychiatric Inventory (NPI)	4 (0 to 11)	8 (3 to 13)	6 (2 to 2)	0.51

Participants had a mean TST of 260.4 min (± 89.9) and a median sleep efficiency of 67% (IQR 48 to 80) (Table 2). There were no significant differences between males and females (Table 2) or amyloid‐positive versus amyloid‐negative participants in (mean) TST, sleep efficiency, or time spent in NREM2 sleep (Tables S6). However, females spent more time than males in NREM3. Women had statistically significantly higher SP density (per 30‐s epoch), power (109 µV2), and median peak SP frequency (11.24 Hz) than men (Table 2 and Table S7). Participants had a mean SO density of 2.7 (± 0.9) per 30‐s epoch, median SO duration of 1 s (IQR 1 to 2), and SO amplitude of 108 µV (IQR 81 to 156). There were no statistically significant differences in SO between men and women (Table 2 and Table S7) or in SP or SO activity by sex and amyloid status (Table S8).

**TABLE 2:** Sleep architecture at baseline.

	Male (*n* = 30)	Female (*n* = 30)	Total (*n* = 60)	*p*
**Sleep architecture**				
Total sleep time (TST) (min)	247.2 ± 93.5	273.6 ± 85.6	260.4 ± 89.9	0.26
Total time in bed (min)	423 (391 to 447)	419 (400 to 443)	421 (392 to 446)	0.63
Sleep efficiency (%)	60 (44 to 80)	69 (50 to 78)	67 (48 to 80)	0.38
Sleep onset latency (min)	17 (9 to 33)	27 (14 to 68)	23 (11 to 57)	0.35
Wake after seep onset (min)	131 (66 to 179)	83 (54 to 136)	101 (58 to 161)	0.11
NREM1 duration (min)	70.1 (47 to 80.5)	44.25 (27.5 to 57)	51 (35.5, 78.25)	0.01
NREM2 duration (min)	86 (63 to 120)	105 (85 to 154)	92 (69 to 138)	0.25
NREM3 duration (min)	43 (24 to 78)	84 (38 to 106)	60 (28 to 91)	0.03
N2+N3 min/TST (%)	55.6 ± 18.2	68.6 ± 17.1	62.1 ± 18.7	0.006
REM duration (min)	27 (14 to 42)	26 (15 to 46)	26 (14 to 44)	0.83
NREM1 (percentage of TST)	30 (23 to 42)	16 (8 to 22)	22 (10 to 34)	0.007
NREM2 (percentage of TST)	37.3 ± 14.7	40.8 ± 13.3	39.0 ± 14.0	0.35
NREM3 (percentage of TST)	16 (10 to 26)	30 (15 to 39)	23 (14 to 36)	0.008
REM (percentage of TST)	10 (7 to 16)	11 (5 to 16)	10 (6 to 16)	0.94

**Sleep spindle (SP)**	**Male (30)**	**Female (30)**	**Total (60)**	***P***
**NREM2+NREM3**				
SP density	0.4 ± 0.3	0.7 ± 0.3	0.5 ± 0.3	0.002
SP duration	0.7 ± 0.1	0.7 ± 0.1	0.7 ± 0.1	0.32
SP power	251 (151 to 300)	280 (211 to 404)	254 (187 to 374)	0.54

**Slow oscillations (SOs)**	**Male (30)**	**Female (30)**	**Total (60)**	***P***
**NREM2+NREM3**				
SO density	2.6 ± 1.0	2.7 ± 0.8	2.7 ± 0.9	0.62
SO duration	1 (1 to 2)	1 (1 to 2)	1 (1 to 2)	0.31
SO peak‐to‐peak amplitude	107 (83 to 144)	116 (80 to 175)	108 (81 to 156)	0.66

At baseline, participants had a mean MMSE score of 22.9 ± 2.4, median ADAS‐Cog subscale total score of 29 (25 to 31), and no significant differences between men and women in all of the cognitive tests, save for a higher score among men in Rey–Osterrieth long‐term visual memory (Table 3). At follow‐up, both men and women had decreased cognitive performance and MMSE scores at 12, 24, and 36 months compared to baseline, with men showing a greater decline in cognitive performance on the MMSE by 36 months, though the difference between men's and women's performance was not statistically significant (Table 3). There were no statistically significant differences between men and women in ADAS‐Cog total scores and short‐ or long‐term verbal memory (CVLT). However, women had statistically significantly poorer performance and greater decline than men in short‐ and long‐term visual memory on the ROCF short‐ and long‐term visual memory tests at baseline and 12 months. Men had higher long‐term visual memory scores (ROCF test) at 12 months than women. Cognition by amyloid status (positive vs negative) can be seen in Table S9.

**TABLE 3:** Change in cognition from baseline to 12, 24, and 36 months.

Full sample	Male (*n* = 30)	Female (*n* = 30)	Total (*n* = 60)	*p*
**Mini‐Mental State Examination (MMSE)**				
Baseline	23.4 ± 2.3	23.0 ± 2.4	23.2 ± 2.4	0.55
12 months	23.0 ± 2.8	21.5 ± 3.9	22.3 ± 3.5	0.01
24 months	20.8 ± 3.6	20.0 ± 3.8	20.4 ± 3.7	0.49
36 months	19.1 ± 5.4	18.7 ± 4.2	18.9 ± 4.7	0.84
Change from baseline to 12 months	−0.18 ± 2.61	−1.41 ± 2.71	−0.81 ± 2.71	0.09
Change from baseline to 24 months	−2.17 ± 3.27	−2.90 ± 3.18	−2.52 ± 3.21	0.46
Change from baseline to 36 months	−4.46 ± 4.97	−3.84 ± 3.88	−4.12 ± 4.34	0.74
**ADAS‐cog**				
Total score, baseline	28 (26 to 32)	29 (25 to 31)	29 (25 to 31)	0.89
Total score, 12 months	27.7 ± 8.4	29.3 ± 7.8	28.5 ± 8.1	0.44
Delayed memory (item 4), baseline	10 (8 to 10)	10 (8 to 10)	10 (8 to 10)	0.38
Delayed memory (item 4), 12 months	9 (8 to 10)	9 (8 to 10)	9 (8 to 10)	0.83
**California Verbal Learning Test**				
Short‐term verbal memory, base	−2 (−2, −1)	−2 (−2, −1)	−2 (−2, −1)	0.83
Short‐term verbal memory, 12 months	−1.6 ± 0.79	−1.8 ± 1.0	−1.7 ± 0.91	0.44
Long‐term verbal memory, base	−1.8 ± .1.0	−1.8 ± 1.33	−1.79 ± 1.33	0.82
Long‐term verbal memory, 12 months	−1.8 ± 0.8	−2.1 ± 1.3	−2.0 ± 1.1	0.23
**Rey–Osterrieth**				
Long‐term visual memory, baseline	5.5 (3.5 to 8.5)	2 (2 to 5)	5 (2 to 7)	0.02
Long‐term visual memory, 12 months	6 (2 to 7.5)	2 (2 to 6)	4 (2 to 7)	0.23
Copy‐recall, baseline	8 (5 to 10)	7 (3 to 9)	7 (3 to 10)	0.9
Copy‐recall, 12 months	6 (2 to 9)	6 (3 to 8)	6 (2 to 8)	0.48

Abbreviations: ADAS, Alzheimer's Disease Assessment Scale, cognitive subscale CVLT, California Verbal Learning Test; MMSE, Mini‐mental state examination; ROCF, Rey–Osterrieth Complex Figure Test.

### 3.1 Biomarkers and cognition

Adjusted for age, sex, and AHI, only the pTau181/Aβ42 ratio at baseline predicted cognitive performance (Table 4). A one‐unit increase in the pTau181/Aβ42 ratio predicted a statistically significant decrease in MMSE scores at 12 months (β = −13.33, 95% confidence interval [CI]: −24.39, −2.26) and 24 months (β = −17.81, 95% CI: −30.17, −5.45). No biomarker or biomarker ratio predicted cognitive performance on the ADAS‐Cog at baseline or 12‐month follow‐up.

**TABLE 4:** AD biomarkers and cognitive performance at baseline, 12, 24, and 36 months.

	MMSE baseline				MMSE at 12 months			
Biomarker	Coefficient	*p*	95% low	Upper	Coefficient	*p*	Lower	Upper
Aβ42	0.00	0.59	−0.003	0.01	0.00	0.92	−0.007	0.01
pTau181	0.01	0.07	−0.001	0.02	−0.00	0.87	−0.02	0.01
Total tau	−0.05	0.93	−1.2	1.21	−1.18	0.12	−2.65	0.29
pTau181/Aβ42	−5.79	0.12	−13.11	1.53	−13.33	0.02	−24.39	−2.26
Total tau/Aβ42	0.00	0.86	−0.003	0.00	−0.00	0.05	−0.006	0.000

	**MMSE at 24 months**				**MMSE at 36 months**			
Biomarker	Coefficient	*p*	95% low	Upper	Coefficient	*p*	Lower	Upper
Aβ42	0.00	0.13	−0.001	0.01	−0.00	0.86	−0.01	0.01
pTau181	0.01	0.59	−0.01	0.02	0.004	0.60	−0.01	0.02
Total tau	−1.06	0.33	−3.17	1.06	0.44	0.73	−2.04	2.93
pTau181/aβ42	−17.81	0.01	−30.16	−5.45	−18.23	0.13	−42.07	5.62
Total tau/aβ42	−0.003	0.21	−0.008	0.002	0.00	0.94	−0.006	0.006

	**ADAS total score baseline**				**ADAS‐total score at 12** **months**			
Biomarker	Coefficient	*p*	95% low	Upper	Coefficient	*p*	Lower	Upper
Aβ42	−0.01	0.37	−0.02	0.01	−0.001	0.91	−0.02	0.01
pTau181	−0.01	0.46	−0.05	0.02	−0.01	0.51	−0.05	0.02
Total tau	0.01	0.07	−0.001	0.02	0.01	0.10	−0.001	0.02
pTau181/aβ42	26.79	0.06	−0.68	54.26	21.03	0.21	−12.06	54.13
Total tau/aβ42	2.94	0.19	−1.42	7.30	2.31	0.29	−1.96	6.57

### 3.2 Sleep and cognition

**FIGURE 2:**
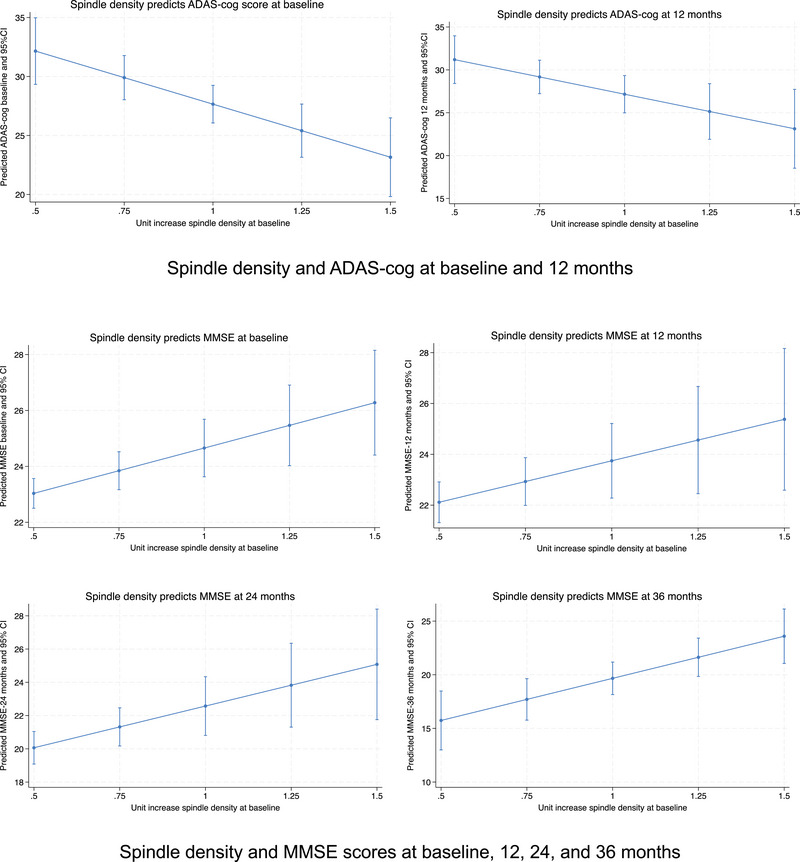
Spindle density predicts cognitive performance on ADAS‐Cog and MMSE.

We found that SP metrics predicted cognitive performance on the  ADAS‐Cog total score at baseline and 12‐month follow‐up (Table 5). A unit increase in SP density predicted a nine‐point decrease in ADAS‐Cog total score at baseline (β = −9.0, *p* = .001) and at 12 months (β = −8.64, *p* = .001) (Figure 2). A one‐unit increase in SP power also predicted decreased ADAS‐Cog scores at baseline (β = −0.03, *p* = .002) and 12 months (β = −0.02, *p* = .009). SP duration predicted a large decrease of 28 points on the ADAS‐Cog at baseline (β = −27.6, *p* = .03) and a 29‐point decrease at 12 months (β = −29.5, *p* = .028), but these did not survive FDR correction (Table 5, and Figure S1). Neither SO density, duration, nor amplitude predicted ADAS‐Cog total score at baseline or 12 months.

**TABLE 5:** GLM regression results, sleep spindle (central channels), slow oscillation (SO) (frontal channels), biomarkers, and cognition.

Biomarkers	Aβ42					pTau181					tTau				
Spindles	Coefficient	SE	*p*	95% CI low	Upper	Coefficient	SE	*p*	95%CI low	Upper	Coefficient	SE	*p*	95%CI low	Upper
SP duration	238.99	571.31	0.676	−880.8	1358.7	**8.25**	**2.15**	**0.001**	**4.04**	**12.45**	391.37	738.72	0.596	−1056.5	1839.2
SP density	87.64	63.79	0.169	−37.39	212.68	1.44	1.76	0.412	−2.01	4.89	−120.7	158.17	0.445	−430.72	189.31
SP energy	**0.250**	**0.08**	**0.003**	**0.09**	**0.41**	−0.01	0.01	0.414	−0.02	0.01	−0.30	0.42	0.472	−1.13	0.52
**SO**															
SO duration	−706.7	326.58	0.030	−1346.7	−66.58	−5.80	5.24	0.268	−16.1	4.46	−151.9	418.95	0.717	−973.1	669.19
SO density	69.33	20.29	**0.001**	29.56	109.11	−0.03	0.07	0.659	−0.17	0.11	−0.047	0.09	0.589	−0.22	0.12
SO peak‐to‐peak amplitude	0.006	0.39	0.989	−0.76	0.77	−0.02	0.02	0.410	−0.06	0.02	0.349	1.10	0.751	−1.81	2.51

	**tTau/aβ42**					**pTau/aβ42**									
**Spindles**	Coefficient	SE	*p*	95%CI low	Upper	**Coefficient**	SE	*p*	95% CI low	Upper					
SP duration	0.507	1.07	0.64	−1.589	2.603	−0.443	0.39	0.261	−1.22	0.33					
SP density	−0.096	0.15	0.53	−0.397	0.204	**−0.081**	**0.03**	**0.009**	**−0.14**	**−0.02**					
SP energy	−0.001	0.00	0.05	−0.001	0.000	**−0.010**	**0.00**	**0.012**	**−0.01**	**0.00**					
**SO**															
SO duration	0.251	0.54	0.64	−0.811	1.31	0.170	0.19	0.37	−0.20	0.54					
SO density	−0.005	0.07	0.94	−0.142	0.13	−0.032	0.02	0.07	−0.07	0.00					
SO ptp amplitude	−0.0001	0.00	0.96	−0.002	0.002	0.0002	0.00	0.4	0.00	0.00					

**Cognition**	**ADAS‐cog at baseline**					**ADAS‐cog at 12 months**					**MMSE at baseline**				
**Spindles**	Coefficient	SE	*p*	95%CI low	Upper	Coefficient	SE	*p*	95%CI low	Upper	Coefficient	SE	*p*	95%CI low	Upper
SP duration	−27.60	12.84	0.032	−52.76	−2.44	−29.45	13.39	0.03	−55.70	−3.21	8.85	6.56	0.177	−4.00	21.70
SP density	**−9.0**	**2.70**	**0.001**	**−14.29**	**−3.7**	**−8.64**	**2.41**	**0.001**	**−13.37**	**−3.91**	**3.24**	**0.94**	**0.001**	**1.41**	**5.08**
SP energy	**−0.03**	**0.01**	**0.002**	**−0.04**	**−0.01**	**−0.02**	**0.01**	**0.01**	**−0.03**	**0.00**	**0.01**	**0.00**	**0.011**	**0.00**	**0.01**
**SO**															
SO duration	25.83	11.83	0.029	2.64	49.01	6.78	11.75	0.56	−16.25	29.81	−5.34	4.83	0.268	−14.80	4.12
SO density	−0.08	1.58	0.959	−3.17	3.01	−0.27	1.22	0.83	−2.67	2.13	−0.34	0.34	0.317	−1.02	0.33
SO peak‐to‐peak amplitude	0.007	0.02	0.757	−0.04	0.05	0.04	0.02	0.08	0.00	0.08	0.03	0.01	0.037	0.00	0.06

	**MMSE 12 months**					**MMSE 24 months**					**MMSE 36 months**				
**Spindles**	Coefficient	SE	*p*	95%CI low	Upper	Coefficient	SE	*p*	95%CI low	Upper	Coefficient	SE	*p*	95%CI low	Upper
SP duration	**15.2**	**5.77**	**0.011**	**3.53**	**26.91**	**22.92**	**10.07**	**0.020**	**3.19**	**42.65**	**34.9**	**11.74**	**0.003**	**11. 87**	**57.91**
SP density	**4.2**	**1.32**	**0.001**	**1.61**	**6.78**	**5.02**	**1.74**	**0.004**	**1.61**	**8.42**	**7.85**	**2.21**	**0.000**	**3.52**	**12.18**
SP energy	**0.02**	**0.00**	**0.001**	**0.01**	**0.02**	0.00	0.00	0.681	0.00	0.01	**0.03**	**0.01**	**0.02**	**0.004**	**0.046**
**SO**															
SO duration	−5.44	5.91	0.357	−17.03	6.14	**−25.38**	**6.89**	**0.000**	**−38.9**	**−11.87**	**−1.45**	**0.48**	**0.01**	**−2.59**	**−0.35**
SO density	−0.78	0.75	0.296	−2.25	0.69	0.65	0.78	0.403	−0.88	2.19	0.07	0.58	0.317	−0.07	0.22
SO peak‐to‐peak amplitude	0.05	0.02	0.034	0.00	0.10	−0.02	0.01	0.144	−0.04	0.01	−0.00	0.00	0.105	−0.07	0.006

	**ROCF copy at baseline**					**ROCF copy at 12 months**					**ROCF long‐term visual memory at baseline**				
**Spindles (SPs)**	Coefficient	SE	*p*	95% CI low	Upper	Coefficient	SE	*p*	95%CI low	Upper	Coefficient	SE	*p*	95%CI low	Upper
SP duration	2.24	1.30	0.08	−0.30	4.79	−0.02	1.20	0.99	−2.36	2.33	**3.34**	**1.39**	**0.01**	**0.62**	**6.07**
SP density	3.63	1.73	0.03	0.24	7.02	0.36	0.25	0.16	−0.14	0.85	0.36	0.28	0.19	−0.18	0.91
SP energy	0.00	0.00	0.72	0.00	0.00	0.001	0.00	0.02	0.00	0.00	0.00	0.00	0.03	0.00	0.00
**SO**															
SO duration	−11.0	8.57	0.2	−27.8	5.81	−10.37	5.56	0.06	−21.3	0.52	−0.32	0.81	0.69	−1.91	1.26
SO density	−0.83	0.58	0.15	−1.97	0.31	0.41	0.52	0.43	−0.60	1.42	−0.07	0.11	0.53	−0.28	0.14
SO peak‐to‐peak amplitude	−0.01	0.01	0.48	−0.03	0.01	0.00	0.01	0.94	−0.02	0.02	0.00	0.00	0.09	−0.01	0.00

	**ROCF long‐term visual memory at 12 months**					**CVLT long‐term verbal memory baseline**					**CVLT long‐term verbal memory at 12 months**				
**Spindles**	Coefficient	SE	*p*	95% CI low	Upper	Coefficient	SE	*p*	95% CI low	Upper	Coefficient	SE	*p*	95%CI low	Upper
SP duration	−3.76	7.23	0.6	−17.94	10.41	−0.54	3.11	0.86	−6.63	5.56	2.34	1.88	0.21	−1.34	6.03
SP density	2.04	1.03	0.04	0.02	4.06	0.29	0.47	0.54	−0.63	1.22	0.68	0.34	0.04	0.02	1.34
SP energy	0.00	0.00	0.58	0.00	0.01	0.00	0.00	0.56	0.00	0.00	**0.001**	**0.00**	**0.01**	**0.00**	**0.00**
**SO**															
SO duration	−7.80	8.23	0.34	−23.94	8.33	1.23	1.85	0.51	−2.40	4.85	−3.44	1.56	0.03	−6.51	−0.38
SO density	0.00	0.00	0.2	−0.01	0.00	−0.04	0.24	0.87	−0.52	0.44	**0.33**	**0.13**	**0.01**	**0.09**	**0.58**
SO peak‐to‐peak amplitude	−0.01	0.01	0.12	−0.03	0.00	0.00	0.00	0.89	−0.01	0.01	0.00	0.00	0.62	−0.01	0.00

*Note*: Results in green are statistically significant. Results in bold remain statistically significant after false discovery rate correction for multiple comparisons.

SP duration, density, and power also predicted increased cognitive performance on the MMSE at baseline and at 12, 24, and 36 months (Table 5). SP density predicted four‐ to eight‐point increases in MMSE score at baseline, 12, 24, and 36 months, with the largest increase predicted by SP density at 36 months (β = 7.85, *p* = .003) (Figure 2). A one‐unit increase in SP density at baseline was also associated with 98.7% lower odds of decreased cognitive performance on the MMSE at 36 months (OR 0.013, 95% CI: 0.001, 0.29), even after adjusting for age, sex, and AHI at baseline. SP duration also predicted increased MMSE scores at 12 months (β = 15.2, *p* = .01), 24 months (β = 22.92, *p* = .02), and 36 months (β = 34.9 *p* = .003) (Table 5 and Figure S1), while SP power predicted small increases in MMSE scores at baseline, 12, and 36 months (Table 5). SO duration was associated with decreased MMSE scores at 24 months (β = −25.4, *p* < .001) and 36 months (β = −2.91, *p* = .001) (Table 5 and Figure S2).

SP density and power predicted increased performance on the copy condition of the ROCF copy‐figure test at baseline and 12 months that were statistically significant but did not survive FDR correction (Table 5). SP duration predicted a statistically significant increase in long‐term visual memory (β = 3.34 *p* = .016) on the ROCF test. However, analyses of SP and SO metrics (density, duration, power, amplitude) in NREM2 alone (Table S4) revealed that SP density also predicted higher Rey copy‐figure score (β = 3.88 *p* = 0.017) ROCF long‐term visual memory tests at baseline (β = 0.67 *p* = 0.019), and long‐term visual memory at 12 months (β = 2.13 *p* = .02). SO amplitude and density in NREM3 also predicted greater performance on the copy‐figure test at 12 months, while SO duration predicted increased long‐term visual performance at 12 months (Table S4).

On the CVLT, SP duration predicted increased short‐term verbal memory performance (β = 0.65 *p* = .01), while SP power (β = 0.001 *p* = .006) and SO density (β = 0.33 *p* = .008) predicted small but statistically significant increases in long‐term verbal memory performance at 12 months. SO duration predicted decreased long‐term verbal memory performance at 12 months (β = −3.44 *p* = .028), but it did not survive FDR correction. As with the ROCF test, analyses of SP duration, density, and power in NREM2 alone and SO metrics in NREM3 alone also resulted in statistically significant increases in short‐ and long‐term memory performance on the CVLT (Table S4).

### 3.3 Sleep and biomarkers

A one‐unit increase of SP power predicted, at a statistically significant level, increased CSF Aβ42 (β = 0.25 *p* = .003) at baseline (Table 5). SO density also predicted a significant increase in Aβ42 (β = 69.33 *p* = .001). SP duration predicted increased CSF pTau181 (β = 7.92, *p* = .001 (Table 5). SP density (β = −0.08, *p* = .01) and power (β = −0.01, *p* = .01) also predicted decreased pTau181/Aβ42. Exploratory analyses of SP metrics in NREM2 only and SO in NREM3 only (Table S1) found several statistically significant associations with Aβ42 (eg, SO density = β 56.34, *p* = .001), pTau181/Aβ42 ratio (SO duration, density, and amplitude), and total‐tau/Aβ42 (eg, SO density β = −0.52, *p* = .001).

A number of statistically significant associations between SP and SO metrics and tau biomarkers were also found among persons who were amyloid‐positive (CSF Aβ42 of < 600 mg/pl) at baseline (Table S1). For example, SO density predicted increased CSF Aβ42 (β = 29.43, *p* = .01) and density (β = 42.73, *p* = .005), while SO duration predicted CSF pTau181 (β = −12.51, *p* = .02) and total tau (β = 4.05, *p* = .01). SP duration, density, and energy also predicted biomarker levels in persons who were amyloid positive at baseline.

### 3.4 Mediation and moderating variables

#### 3.4.1 Sleep SPs and SOs mediate the effect of pTau181/aβ42 on cognition

**FIGURE 3:**
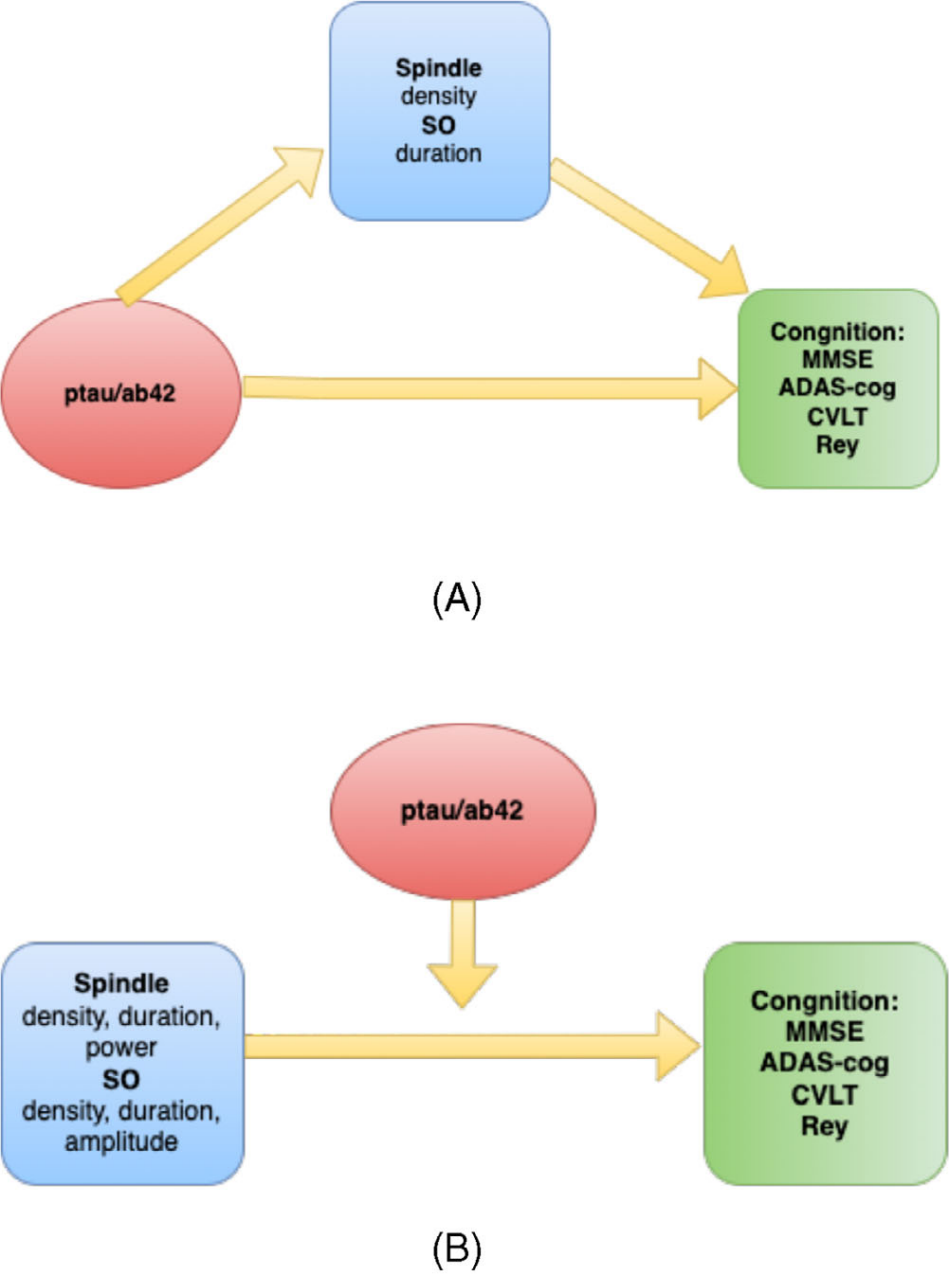
Spindle and SO activity mediates effect of pTau181/aβ42 on cognition (A), while pTau181/aβ42 ratio moderates effects of spindle and SO characteristics on cognition (B).

Sleep SP activity had a significant mediating effect on the relationship between pTau181/aβ42 and cognitive performance on the MMSE and ADAS‐Cog (Figure 3). SP density mediated 41% of the total effect of pTau181/Aβ42 on MMSE scores (indirect effect 69.4% of the direct effect), and SP density mediated 59% of the total effect of pTau181/Aβ42 on ADAS‐Cog scores. This suggests that a large part of the effect of pTau181/Aβ42 on cognition may be due to the mediating role of SPs.

SO duration had a significant mediating effect on the relationship between pTau181/Aβ42 and cognitive performance on the MMSE. The pTau181/aβ42 ratio had a significant negative effect on MMSE scores which is suppressed by SO duration. The proportion of the total effect that was mediated by SO duration is −0.820, indicating a suppression effect. The ratio of the indirect to direct effect was ‐0.450, suggesting a significant mediating role and pathway for SO duration in the relationship between pTau181/Aβ42 ratio and MMSE scores.

#### 3.4.2 PTau181/aβ42 moderates the effect of SPs and SOs on cognition

PTau181/aβ42 had a moderating, but not mediating, effect on the relationship between sleep SPs and cognition (Figure 3). There were statistically significant interactions between pTau181/aβ42 and SP density and power on the ADAS‐Cog, CVLT long‐term verbal memory, MMSE, and ROCF long‐term visual memory and copy‐figure scores. For example, the effect of increased SP density on MMSE scores (β = 35.02, *p* = .001), CVLT long‐term memory at 12 months (β = 16.4, *p* = .001), ROCF long‐term visual memory (β = 16.4, *p* = .001) and copy‐figure (β = 62.0, *p* = .03) increases as pTau181/aβ42 ratio increased. The effect of SP power on ADAS‐Cog scores increased as pTau181/aβ42 increased (β = 0.20, *p* = .013).

Conversely, pTau181/aβ42 had a statistically significant (*p* = .008), negative moderating effect on the relationship between SP duration and power on CVLT long‐term verbal memory (power β = −0.15, *p* = .001), MMSE (duration β = −269.7, *p* = .01, power β = −0.2, *p* = .0001), and ROCF long‐term visual memory (duration β = −172.6 *p* = .006, power β = −0.9, *p* = .003) and copy‐figure (β = −172.7, *p* = .006) scores. The effect of SP duration or power on MMSE scores decreased as pTau181/aβ42 increased. PTau181/aβ42 also had a moderating effect on the relationship between SP duration and power and ADAS‐Cog scores at 12 months. For example, the effect of increased SP power on ADAS scores at 12 months increased as pTau181/aβ42 ratio increased.

PTau181/aβ42 had a statistically significant moderating effect on the effects of SO on cognition scores. For example, pTau181/aβ42 had a significant and additive moderating effect on the effects of SO duration on ADAS‐Cog score (β = 81.1, *p* = .01), SO density on the Rey copy‐figure test (β = 15.3, *p* = .002) and SO amplitude on the MMSE (β = 2.68, *p* = .005). The effects of SO density and amplitude on cognition increased with higher levels of pTau181/aβ42.

## 4 DISCUSSION

SP and SO activity at baseline predicted key biomarkers for AD and cognitive performance from baseline through 36 months. We also found associations between SP characteristics, biomarkers, and cognition in persons with AD previously unreported, to the best of our knowledge. Our findings have important implications for clinical care in the early stages of AD and the development of sleep‐based treatment strategies to delay AD progression.^6^, ^19^ Delaying the onset of clinical symptoms by just 5 years may reduce treatment costs by 40% and add 2.7 additional life years (5 disease‐free years) for persons with AD.^65^

SP density had previously been shown to predict better cognitive performance in healthy older adults, but not long‐term cognitive performance in persons with AD.^3^, ^20^, ^31^, ^32^, ^49^ We found that increased SP density predicted large, clinically significant decreases in ADAS‐Cog scores at baseline and 12 months (>10 points) and higher MMSE scores from baseline through 36 months (three to five points) in persons with mild to moderate AD. Lower ADAS‐Cog scores indicate better cognitive performance, while higher MMSE scores are associated with better cognition. Changes of ≥4 points on the ADAS‐Cog and ≥3 points on the MMSE are clinically important.^36^ The ADAS‐Cog also assesses cognitive and behavioral domains most affected by AD and is a gold standard for assessing the efficacy of treatments targeting dementia.^35^

Most research has investigated the effects of SP density, not duration or power, on cognition or disease progression in AD.^16^ We found higher SP duration and power also predicted better cognitive performance over time in persons with AD. SP duration predicted increased MMSE scores at 12, 24, and 36 months (15 to 34 points). Increased SP density and duration were also associated with significantly lower odds of decreased cognitive performance on the MMSE at 36 months.

Sleep SPs play important roles in cognition, memory consolidation, and synaptic plasticity during sleep.^44^, ^66^, ^67^ SPs are particularly involved in the transfer of information from short‐term memory in the hippocampus to long‐term memory in the neocortex, which is critical for both declarative and procedural memory.^44^, ^66^, ^67^ Higher SP activity has been linked with better cognitive performance.^3^ The synchronization (cross‐frequency coupling) of NREM SO and SPs also plays an important role in sleep‐related memory consolidation in older adults.^44^, ^68^

However, SP density, duration, frequency, and cross‐frequency coupling decrease with normal aging and are associated with the cognitive decline and impaired memory experienced by older adults.^3^, ^66^ Greater reductions in SP density and duration are found in MCI and AD.^5^, ^31^, ^32^, ^52^ This may be due to several factors.^13^ SP density is sensitive to prior learning experience, which may be reduced in persons with AD.^49^ Additionally, physiological changes associated with AD progression reduce synaptic and dendritic integrity and lead to neuronal hyperexcitability and hypersynchronous network activity, impairing sleep SP generation and memory consolidation.^69^ Alzheimer's pathology can also drive neuronal degeneration in areas crucial for sleep regulation, SP activity, or memory consolidation, such as cholinergic neurons in the basal forebrain or noradrenergic neurons in the locus coeruleus.^70^ Cortical atrophy and loss of gray matter volume in the hippocampus, precuneus, amygdala, and cingulate gyrus may also be associated with declines in SP and SO activity.^9^

The accumulation of pTau and Aβ may also influence reductions in SP activity and cognition in older adults.^7^, ^20^ Tau plays a leading role in the formation of neurofibrillary tangles(NFTs), which are hallmarks of AD and are strongly correlated with cognitive decline.^6^ During early AD, pTau181 accumulates in areas associated with arousal and sleep regulation and may play a role in sleep‐wake disruptions, disrupting SP activity and impairing memory consolidation.^71^ Tau pathology in the medial temporal lobe is associated with reductions in hippocampal ripples and the synchronization of SP‐ripple events, also impairing memory consolidation during sleep.^72^

Both SP density and tau in the brain have been associated with cognition in older adults, but little research had investigated their associations in persons already showing clinical AD symptoms.^16^ We found that SP activity was associated with, and predicted, CSF pTau181, tTau, Aβ42, and cognitive performance in persons with AD. This is important, given the significant correlations between tau accumulation, neurodegeneration, cognitive impairment, and dementia in AD.^6^, ^72^

Few researchers have investigated the association between SPs and Aβ42 accumulation in AD.^11^, ^13^, ^26^, ^27^, ^28^, ^29^, ^73^, ^74^ We found that SP activity predicted Aβ42, pTau181/Aβ42, and tTau/Aβ42. In fact, we found that SP activity more frequently predicted Aβ42, tau, and cognitive performance than SO. Nevertheless, SO density predicted a significant increase in Aβ42 at baseline and greater long‐term verbal memory performance on the CVLT. SO duration also predicted decreased CSF‐pTau181 and increased performance on the ROCF test.

An unexpected finding was that longer SO duration predicted reduced long‐term verbal memory at 12 months (CVLT) and poorer MMSE scores at 24 and 36 months. Previous research correlated reduced SWA with increased cognitive impairment in older adults.^75^ Our findings may be driven by AD‐related sleep impairments leading to longer depolarization during SWS and reduced cognitive performance over time.

Increased SO duration was also associated with lower CSF Aβ42 (higher amyloid pathology), which could result from disease progression or be accelerated by reduced SWS over time.

Our findings for SPs, SOs, and cognition likely reflect complex interactions between sleep, Aβ42, tau, and cognition in AD.^28^ Indeed, we found that pTau181/Aβ42 moderated the effects of SP and SO activity on cognition, while SPs and SOs mediated the effects of pTau181/Aβ42 on cognition (Figure 3). Mander et al. (2015) found that sleep mediated the effect of Aβ42 on reduced memory.^11^ Zavecz et al. (2023) also found that NREM SWA significantly suppressed the effect of Aβ status on memory function, particularly in persons with high Aβ burden.^24^

Potential pathways may include orexinergic and glymphatic system activity during NREM sleep, which helps clear AD biomarkers.^76^ Orexin levels are higher in moderate to advanced AD, affect Aβ42 deposition, and can be involved in sleep‐wake cycle dysregulation.^76^ Impaired NREM sleep also increases Aβ42 deposition, which can impair NREM sleep in turn, influencing cognition.^28^ Aβ42 may also reduce NREM SO generation, decreasing Aβ42 clearance, further accelerating AD.^29^

Another intriguing finding was higher SP activity among women in our sample. Sex‐based differences in SP activity have not been investigated extensively in AD. However, higher SP density, particularly in fast SPs (>12 Hz), SP power, and power in the high‐frequency portion of the sigma band (13 to 15 Hz) haven been reported in healthy older women than men.^15^ Greater NREM sleep disruption and up to 50% less SWS have been found in older men than women.^77^, ^78^ These could underlie greater alterations in SP activity in men than women. We also found no statistically significant sex‐based differences in SO activity, though these have been reported for SO and SWA in healthy adults.^77^, ^78^, ^79^ This may be related to sex‐based differences in the development and progression of AD and its effect on sleep physiology, but these have been underinvestigated and need further research, given our findings.^80^

### 4.1 Implications and future research

Our findings support previous suggestions that SP and SO activity can act as predictive, non‐invasive biomarkers for AD progression and provide therapeutic targets for cognitive decline. This is important, given that there are currently few disease‐modifying treatments for AD, and it can be difficult to identify persons for treatment before Aβ burden is too high.^81^ Accessible, in‐home, ambulatory EEG may also improve the feasibility of overnight PSG for persons with AD. Interventions targeting SWA and memory consolidation in persons with MCI or AD, such as transcranial direct‐current or acoustic stimulation, have also yielded promising results.^82^, ^83^

Lifestyle‐based interventions can also enhance sleep in AD. Exercise targets sleep physiology and SWA,^84^, ^85^ can protect against functional and cognitive decline,^86^ decreases levels of biomarkers associated with cognitive decline, and increases those linked to brain health.^87^ Our recent meta‐analysis of exercise interventions targeting sleep in older adults with MCI or AD found moderate‐ to high‐quality evidence for the beneficial effects of exercise on sleep, including SWS.^85^

### 4.2 Strengths and limitations

Our study has several strengths. Our data were collected prospectively in a sex‐balanced cohort of persons with high Aβ42 burden and clinical symptoms consistent with mild to moderate AD using standardized procedures at a center with expertise in AD/ADRD. We also have comprehensive data for cognition capturing a range of cognitive domains.

We controlled for important confounding factors, including age and OSA, while avoiding overfitting of our regression models. AD and OSA share mechanisms and features, including altered sleep architecture, AHI, and reduced brain health.^8^, ^88^ Increasing age is also associated with changes in sleep and some AD biomarkers.^1^ We adjusted for multiple comparisons, reducing risk of Type 1 error.

However, observational studies can only imply associations, not causation, between sleep, biomarkers, and cognition, and we had no comparison group of healthy older adults. However, the study sought to follow the cognitive evolution of persons with AD, and associations between sleep physiology, biomarkers, and cognition in healthy older adults have been explored extensively.^3^, ^20^, ^31^, ^32^, ^49^, ^75^ Participants also underwent PSG only at baseline, without a preceding accommodation night, so we cannot rule out first‐night effects. Overnight PSG with persons with AD carries unique challenges, making this difficult to accomplish. We also cannot rule out the effects of progressive changes in biomarkers or of sleep on cognitive performance as participants’ AD progressed. This would constitute important additional data for future studies to collect, along with PET, to investigate topographic associations between alterations in SPs and anatomical distribution of NFTs.

Our novel findings demonstrate that reduced NREM SO and SO activity constitute complementary, predictive, and non‐invasive biomarkers for AD pathology and progression.^16^, ^50^ Sleep is an important modifiable risk factor for cognitive decline and the progression of AD/ADRD.^1^ Sleep‐based biomarkers for AD, such as NREM SP and SO, may provide novel therapeutic targets for interventions designed to slow the progression of AD symptoms.^6^, ^19^

## CONFLICT OF INTEREST STATEMENT

The authors have no conflicts of interests to declare. Author disclosures are available in the supporting information.

## CONSENT STATEMENT

The study from which these data were analyzed, “The Role of Hypoxia and Sleep Fragmentation in Alzheimer's Disease,” was approved by the Institutional Review Board of Hospital Arnau de Vilanova de Lleida (CE‐1218) and conducted according to the ethical standards of the Declaration of Helsinki (1964) and its later amendments. The protocol was registered (ClinicalTrials.gov NCT02814045).

## Supporting information



Supporting Information

Supporting Information

Supporting Information

Supporting Information

Supporting Information

Supporting Information

Supporting Information

Supporting Information

Supporting Information

Supporting Information

Supporting Information

Supporting Information
