# Supplementary material for: Sleep spindles and slow oscillations predict cognition and biomarkers of neurodegeneration in mild to moderate Alzheimer's disease
Source: Alzheimers Dement. 2025 Jan 29;21(2):e14424. doi: 10.1002/alz.14424 (PMC11848347; doi:10.1002/alz.14424)
Supplement: Supplementary file 2 — Supporting Information [file ALZ-21-e14424-s004.docx]

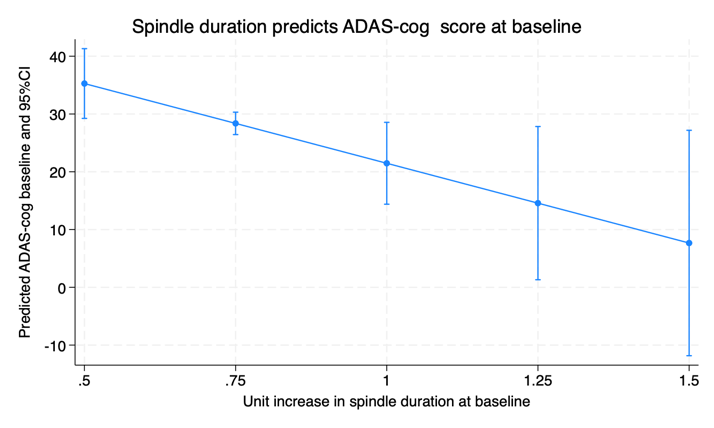

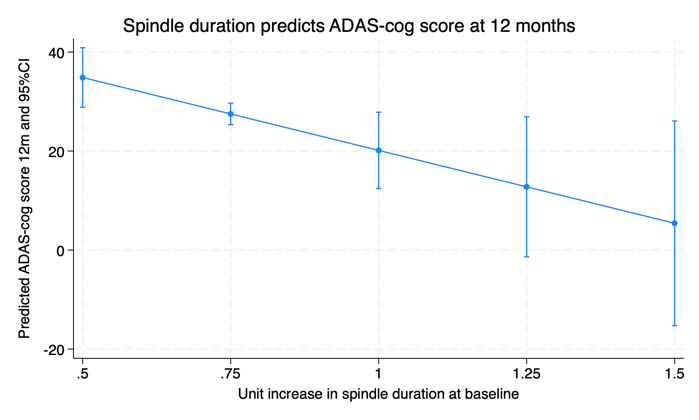


Spindle duration predicts ADAS-cog scores


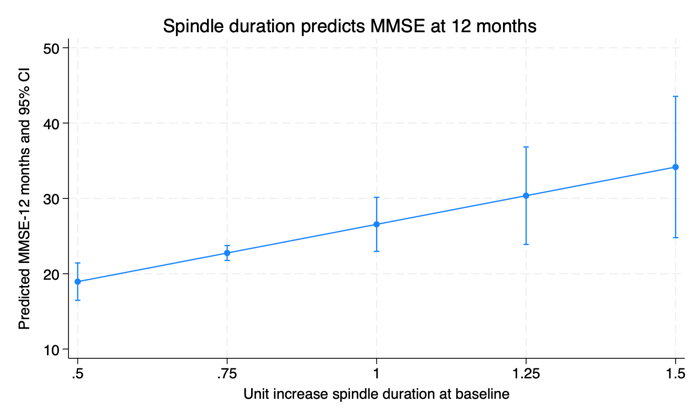

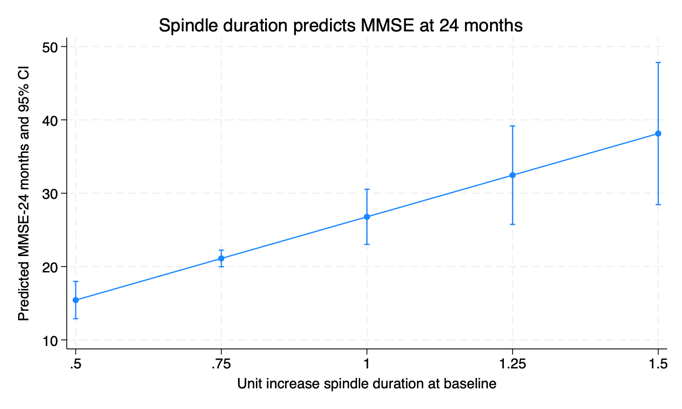


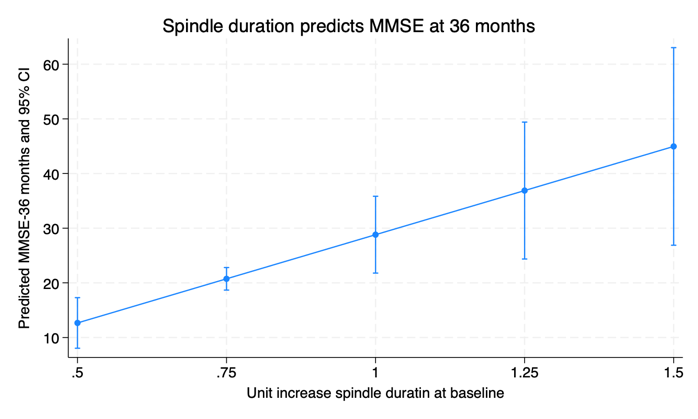


**Supplementary material Figure S1:** Spindle duration at baseline predicts cognitive performance on the ADAS-cog and MMSE (margins plot)
