# Supplementary material for: Sleep spindles and slow oscillations predict cognition and biomarkers of neurodegeneration in mild to moderate Alzheimer's disease
Source: Alzheimers Dement. 2025 Jan 29;21(2):e14424. doi: 10.1002/alz.14424 (PMC11848347; doi:10.1002/alz.14424)
Supplement: Supplementary file 3 — Supporting Information [file ALZ-21-e14424-s005.docx]

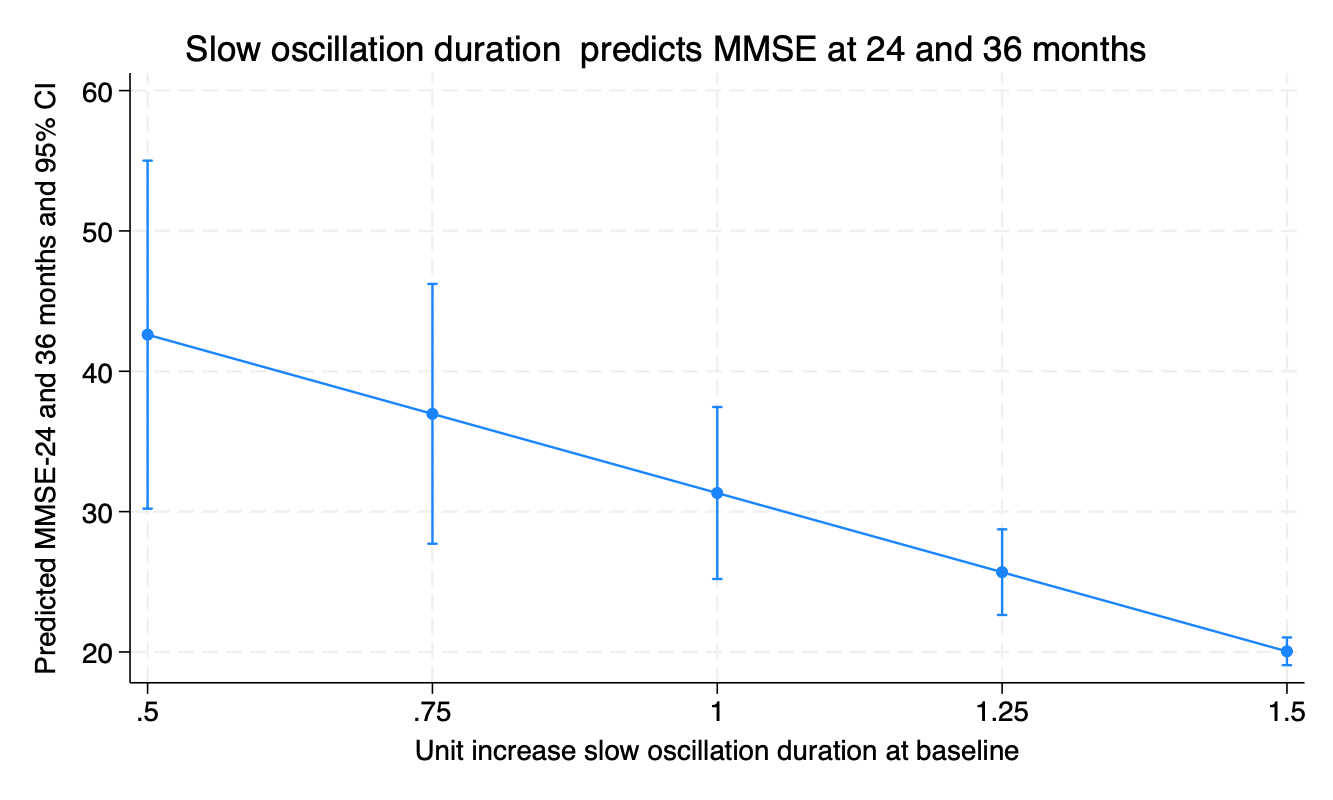


**Supplementary material Figure S2:** Slow oscillation duration predicts MMSE at 24 and 36 months (margins plot)
