# Supplementary material for: Sleep spindles and slow oscillations predict cognition and biomarkers of neurodegeneration in mild to moderate Alzheimer's disease
Source: Alzheimers Dement. 2025 Jan 29;21(2):e14424. doi: 10.1002/alz.14424 (PMC11848347; doi:10.1002/alz.14424)
Supplement: Supplementary file 8 — Supporting Information [file ALZ-21-e14424-s009.docx]

**Supplementary Table S5** : Characteristics of participants with Aβ42<600 pg/ml at baseline

| **Amyloid + (<600 pg/ml) only** | n=19 (51.3%) | n=18(48.7%) | n=37 | 0.84 |
| --- | --- | --- | --- | --- |
| age | 75.6 ±5.74 | 73.3 ±5.4 | 74.5±5.6 | 0.22 |
| bmi | 27.4 ±3.9 | 28.2 ±4.8 | 27.8 ±4.3 | 0.58 |
| depression | 5 (26.3%) | 9 (50%) | 14 (37%) | 0.14 |
| diabetes | 4 (21%) | 2 (11%) | 6 (16 %) | 0.42 |
| **education** (≥ high school) | 4 (21.1%) | 3 (16.7%) | 7 (19%) | 0.73 |
| 0: no formal education | 2 (10.5%) | 2 (11.1%) | 4 (10.8%) |  |
| 1. Primary school | 13 (68.4%) | 13 (72.2%) | 26 (70.3%) |  |
| 2. High school | 3 (15.79%) | 2 (11.1%) | 5 (13.5%) |  |
| 3. University | 1 (5.3%) | 1 (5.6%) | 2 (5.4%) |  |
| **Apnoea hypoxia index** (n/hrTST) | 39.58 ±20.24 | 33.07 ±24.39 | 36.42 ±24.4 | 0.38 |
| **AD Drugs** | 18 (95%) | 16 (89%) | 34 (92%) | 0.51 |
| none | 1 (5.3%) | 2 (11.1%) | 3(8.1%) |  |
| Rivastigmina | 5 (26.3%) | 5 (27.8) | 10 (27%) |  |
| Donepezil | 13 (68.4%) | 8 (44.4%) | 21 (56.8%) |  |
| Memantine | 0 | 3 (6.7%) | 3 (8.1%) |  |
| **Lab values-biomarkers (pg/ml)** |  |  |  |  |
| Aβ42, pg/ml | 450.53 ±88.0 | 460.22 ±84.07 | 455.24 ±85.05 | 0.73 |
| CSF p-tau pg/ml | 71.53 ±35.24 | 4.21 ±27.98 | 77.87 ±32.0 | 0.24 |
| plasma tau, pgml | 2.21 ±1.0 | 2.89 ±,93 | 2.55 ±1.01 | 0.05 |
| CSF total tau, pg/ml | 483.0 ±332.12 | 594.67 ±249.71 | 537.33 ±296.31 | 0.26 |
| CSF p-tau/ Aβ42 ratio pg/ml | 0.16 ±0.075 | 0.189 ±0.072 | 0.174 ±0.74 | 0.25 |
| CSF total-tau/ Aβ42 ratio pg/ml | 1.09 ± 0.75 | 1.34 ± 0.63 | 1.21 ± 0.70 | 0.29 |
| **Cognition, mental health** |  |  |  |  |
| ADAS-cog total score | 30 (25-33) | 29.5 (25-35) | 30 (25-34) | 0.49 |
| ADAS-cog delayed memory | 30 (25-33) | 29.5 (25-35) | 30 (25-34) | 0.42 |
| Cornell Scale (CSDD) | 7.4 ±6.3 | 7.6 ±5.4 | 7.46 ±5.8 | 0.88 |
| MMSE | 23.2 ±2.5 | 22.6 ±2.4 | 22.9 ±2.4 | 0.49 |
| **By amyloid status at baseline** | **<600 pg/ml** (n 23) | **>600 pg/ml** (n 37) | **p value** |  |
| Age | 75.09 (4.04) | 74.51 (5.63) | 0.67 |  |
| Female | 12 (52.2%) | 18 (48.6%) | 0.79 |  |
| BMI | 28.32 (5.98) | 27.81 (4.32) | 0.70 |  |
| **Smoking history** |  |  |  |  |
| 0. never | 18 (78.3%) | 29 (78.4%) | 0.94 |  |
| 1. current | 1 (4.3%) | 1 (2.7%) |  |  |
| 2. former (>6 mths ago) | 4 (17.4%) | 7 (18.9%) |  |  |
| OSA diagnosis | 15 (78.9%) | 32 (100.0%) | 0.009 |  |
| **Apnoea hypoxia index** (n/hrTST) | 32.6 (16.4-56.3) | 23.4 (11.2-47.3) | 0.17 |  |
| 0-4.9 | 0 | 3 (13%) |  |  |
| 5-14.99 | 7 (18.9%) | 4 (17.4%) |  |  |
| 15-29.99 | 11 (29.7%) | 8 (34.8%) |  |  |
| ≥ 30 | 19 (51.4%) | 8 (34.8%) |  |  |
| **Cognition, mental health** |  |  |  |  |
| ADAS-cog total score | 27.43 (5.64) | 30.36 (8.25) | 0.16 |  |
| ADAS-cog delayed memory | 7 (6-8) | 7(6-7) | 0.15 |  |
| Cornell Scale (CSDD) | 7 (2-11) | 7 (3-14) | 0.96 |  |
| MMSE baseline | 23.65 (2.29) | 22.49 (3.77) | 0.19 |  |
| NPI | 9.10 (16.14) | 10.75 (13.12) | 0.68 |  |
| **Lab values-biomarkers (pg/ml)** |  |  |  |  |
| Aβ42, pg/ml | 737.27 (145.44) | 455.24 (85.05) | <0.001 |  |
| CSF p-tau pg/ml | 76.23 (30.90) | 88.82 (73.70) | 0.53 |  |
| plasma tau, pgml | 2.52 (1.29) | 2.55 (1.01) | 0.92 |  |
| CSF total tau, pg/ml | 558.07 (264.23) | 537.33 (296.31) | 0.82 |  |
| CSF p-tau/ Aβ42 ratio pg/ml | 0.17 ± 0.74 | 0.11 ± 0.52 | 0.72 |  |
| CSF total-tau/ Aβ42 ratio pg/ml | 1.21 ± 0.70 | 0.80 ± 0.70 | 0.04 |  |
