# Supplementary material for: Sleep spindles and slow oscillations predict cognition and biomarkers of neurodegeneration in mild to moderate Alzheimer's disease
Source: Alzheimers Dement. 2025 Jan 29;21(2):e14424. doi: 10.1002/alz.14424 (PMC11848347; doi:10.1002/alz.14424)
Supplement: Supplementary file 9 — Supporting Information [file ALZ-21-e14424-s008.docx]

|  | **Amyloid at baseline** | | |
| --- | --- | --- | --- |
|  | **<600 (SD)** | **>600 (SD)** | **Test (p)** |
|  | N=23 | N=37 |  |
| TST (min) | 244.80 (78.78) | 270.11 (95.88) | 0.293 |
| SE (%) | 58.22 (18.91) | 65.08 (22.85) | 0.240 |
| WASO (min) | 129.18 (59.79) | 108.59 (77.73) | 0.299 |
| SOL (min) | 44.09 (44.48) | 36.66 (52.12) | 0.573 |
| NREM 1 (min) | 65.91 (33.89) | 51.45 (31.33) | 0.097 |
| NREM2 (min) | 97.38 (39.04) | 110.40 (68.80) | 0.412 |
| NREM3 (min) | 52.04 (40.48) | 73.04 (50.09) | 0.096 |
| N2+N3 dur (min) | 149.43 (61.58) | 183.44 (97.06) | 0.139 |
| N2N3/TST (%) | 60 % (16) | 64 % (20) | 0.423 |
| REM (min) | 29.47 (24.15) | 34.53 (31.73) | 0.515 |
| REM_latency | 151.04 (94.44) | 182.10 (93.12) | 0.255 |
| N1 % | 29.67 (18.62) | 24.00 (20.04) | 0.279 |
| N2 % | 39.65 (11.78) | 38.66 (15.35) | 0.793 |
| N3 % | 19.97 (13.20) | 25.09 (15.06) | 0.185 |
| REM % | 10.73 (7.22) | 12.24 (10.41) | 0.545 |
| N1 latency (min) | 44.09 (44.48) | 37.71 (51.57) | 0.626 |
| N2 latency (min) | 49.48 (43.68) | 47.55 (60.71) | 0.895 |
| N3 latency (min) | 83.91 (65.36) | 77.33 (72.37) | 0.724 |
| REM latency (min) | 142.07 (97.58) | 166.93 (102.63) | 0.381 |
| AHI (#/hr TST) | 29.94 (24.67) | 36.42 (22.29) | 0.304 |

**Supplementary Table S6:** Sleep architecture by amyloid status

There were no statistically significant differences in total sleep time, sleep efficiency, or sleep macro-architecture between persons with amyloid < than 600 pg/ml (AB+) or > 600 pg/ml (AB-) (table 3) .
