# Supplementary material for: Sleep spindles and slow oscillations predict cognition and biomarkers of neurodegeneration in mild to moderate Alzheimer's disease
Source: Alzheimers Dement. 2025 Jan 29;21(2):e14424. doi: 10.1002/alz.14424 (PMC11848347; doi:10.1002/alz.14424)
Supplement: Supplementary file 10 — Supporting Information [file ALZ-21-e14424-s007.docx]

| **Sleep Spindles** | Men (30) | Women (30) | All | p |
| --- | --- | --- | --- | --- |
| **NREM2** |  |  |  |  |
| SP density | 0.4 ±0.3 | 0.7 ±0.3 | 0.6 ±0.3 | 0.002 |
| SP duration | 1 (1-1) | 1 (1-1) | 1 (1-1) | 0.62 |
| SP power | 257 (151-312) | 272 (221-408) | 265 (188-381) | 0.63 |
| **NREM3** |  |  |  |  |
| SP density | 0 (0-1) | 1 (0-1) | 0 (0-1) | 0.006 |
| SP duration | 1 0.7 ±0.1 | 1 0.7 ±0.1 | 1 0.7 ±0.1 | 0.29 |
| SP power | 223 (143-328) | 315 (199-386) | 243 (179-349) | 0.26 |
| **Slow Oscillations** |  |  |  |  |
| **NREM2** |  |  |  |  |
| SO density | 2.6 ±1.0 | 2.6 ±0.8 | 2.6 ±0.9 | 0.87 |
| SO duration | 1 (1-2) | 1 (1-1) | 1 (1-2) | 0.16 |
| SO ptp amplitude | 104 (79-137) | 114 (76-164) | 107 (77-145) | 0.77 |
| **NREM3** |  |  |  |  |
| SO density | 3 (2-3) | 3 (2-4) | 3 (2-4) | 0.36 |
| SO duration | 2 (1-2) | 2 (1-2) | 2 (1-2) | 0.62 |
| SO ptp amplitude | 114.6 ±65.7 | 125.9 ±66.2 | 120.2 ±65.6 | 0.51 |

**Supplementary Material Table S7:** NREM2 and NREM2 microarchitecture, whole sample

There were no statistically significant differences in SO between men and women, save for higher SO count in NREM among women than men.
