# Supplementary material for: Sleep spindles and slow oscillations predict cognition and biomarkers of neurodegeneration in mild to moderate Alzheimer's disease
Source: Alzheimers Dement. 2025 Jan 29;21(2):e14424. doi: 10.1002/alz.14424 (PMC11848347; doi:10.1002/alz.14424)
Supplement: Supplementary file 11 — Supporting Information [file ALZ-21-e14424-s001.docx]

| **amyloid + (<600pg/ml)** | **Male (n=19)** | **Female (n=18)** | **Total (n=37)** | **p** |
| --- | --- | --- | --- | --- |
| Total sleep time- TST (min) | 261.6 (172-328) | 302.8 (258.5-359) | 274.1 (218-343.5) | 0.10 |
| Total time in bed (min) | 405.2 ±35.9 | 424.2 ±26.6 | 414.41 ±32.7 | 0.08 |
| Sleep efficiency (%) | 61.8 (50.6-80.3) | 70.95 (59.4-82.6) | 70.7 (53.9-80.8) | 0.24 |
| Sleep onset latency (min) | 17.6 (6-27.9) | 23.7 (11.3-67.9) | 20.3 (10.8-33) | 0.57 |
| Wake after seep onset (min) | 146 (56.5- 193.3) | 71.55 (39.8-100.7) | 79.4 (52.1-161.5) | 0.08 |
| NREM 1 (min) | 31.1 (13.4-46.7) | 12.45 (8.4-20.5) | 17.6 (9.3-32) | 0.06 |
| NREM2 (min) | 97.7 ±74 | 123.8 ±62.1 | 110.40 ±68.80 | 0.14 |
| NREM3 (min) | 53.82 ±49.8 | 93.3 ±42.9 | 73.04 ±50.09 | 0.003 |
| N2+N3min/TST (%) | 55.5 ±21.6 | 72.2 ±14.8 | 63.1 % (20.23) | 0.01 |
| REM (min) | 30 (9-44) | 25 (16.5-45.5) | 28.5 (14.5-44) | 0.95 |
| NREM1 (% of TST) | 31.1 (13.4-46.7) | 12.45 (8.4-20.5) | 17.6 (9.3-32) | 0.008 |
| NREM2 (% of TST) | 37.06 ±17.04 | 40.34 ±17.04 | 38.66 ±15.35 | 0.52 |
| NREM3 (% of TST) | 25.09 ±14.92 | 32.17 ±11.88 | 25.09 ±15.06 | 0.004 |
| REM (% of TST) | 9 (3.3-17.3) | 11.55 (5.4-14.1) | 11.3 (5.4-16.2) | 0.72 |
| **Sleep spindle (SP)** |  |  |  |  |
| **NREM2+NREM3** |  |  |  |  |
| SP density | 0.442 ±0.28 | 0.595 ±0.252 | 0.517 ±0.274 | 0.09 |
| SP duration | 0.697 ±0.758 | 0.7 ±0.048 | 0.699 ±0.063 | 0.88 |
| SP power | 234.06 ±181.69 | 300.16 ±112.66 | 266.22 ±153.69 | 0.20 |
|  |  |  |  |  |
| **NREM2** |  |  |  |  |
| SP density | 0.454 ±0.288 | 0.626 ±0.233 | 0.538 ±0.276 | 0.06 |
| SP duration | 0.7 (0.66-0.73) | 0.71 (0.67-0.72) | 0.071(0.66-0.72) | 0.89 |
| SP power | 196.39 (125.56-269.88) | 279.64 (210.9-374) | 241.91 ((161.68-304.23) | 0.20 |
| **Slow Oscillations (SO)** |  |  |  |  |
| **NREM2+NREM3** |  |  |  |  |
| SO density | 2.63 ±0.99 | 2.75 ±0.77 | 2.48 ±0.94 | 0.47 |
| SO duration | 1.49 (1.46-1.55) | 1.51 (1.47-1.53) | 1.5 (1.46-1.54) | 0.84 |
| SO ptp amplitude | 117.473 ±46.7 | 111.79 ±47.99 | 114.68 ±46.74 | 0.72 |
| **NREM3** |  |  |  |  |
| SO **density** | 2.29 ±1.25 | 2.63 ±1.11 | 2.46 ±1.81 | 0.39 |
| SO duration | 1.49 (1.46-1.55) | 1.51 (1.5-1.53) | 1.50 (1.46-1.54) | 0.63 |
| SO ptp **amplitude** | 106.44 ±66.87 | 116.11 ±62.88 | 111.14 ±64.24 | 0.65 |

**Supplementary material Table S8.** Sleep microarchitecture among persons with amyloid beta <600 pg/ml at baseline

Amyloid-positive women spent more time in NREM3, both in minutes and as the percentage of their total sleep time (p= 0.003) and NREM2 and NREM3 as a percentage of total sleep time (p=0.006) than Aβ+ men, who spent less time in NREM3 and had statistically greater percentage of NREM1 sleep out of their total sleep time than women.
