# Supplementary material for: Sleep spindles and slow oscillations predict cognition and biomarkers of neurodegeneration in mild to moderate Alzheimer's disease
Source: Alzheimers Dement. 2025 Jan 29;21(2):e14424. doi: 10.1002/alz.14424 (PMC11848347; doi:10.1002/alz.14424)
Supplement: Supplementary file 12 — Supporting Information [file ALZ-21-e14424-s012.docx]

| **amyloid + (<600 pg/ml)** | **n=19** | **n=18** | **total** | **p value** |
| --- | --- | --- | --- | --- |
| **Mini mental stage exam (MMSE)** |  |  |  |  |
| baseline | 23.2 ±2.5 | 22.6 ±2.4 | 22.9 ±2.4 | 0.49 |
| 12 months | 22.9 ±2.7 | 21.2 ±3.4 | 22.1 ±3.1 | 0.01 |
| 24 months | 21.5 ±3.9 | 18.6±3.5 | 20.2 ±3.9 | 0.04 |
| 36 months | 20.6 ±4.3 | 17.7 ±4.4 | 19.3 ±4.5 | 0.22 |
| Change from base to 36 months | -2.89 ± 3.26 | -4.57 ±4.99 | -3.63 ±4.1 | 0.43 |
| **ADAS-cog** |  |  |  |  |
| total score | 30 (25-33) | 29.5 (25-35) | 30 (25-34) | 0.49 |
| total score 12m | 30 (25-33) | 29.5 (25-35) | 30 (25-34) | 0.42 |
| **California Verbal Learning Test** |  |  |  |  |
| Short-term verbal memory, base | -1.7 ± 0.82 | -1.4 ± 1.7 | -1.6 ± 1.3 | 0.59 |
| Short-term verbal memory 12m | -1.7 ± 0.75 | -1.9 ± 1.2 | -1.8 ± 1 | 0.51 |
| Long term verbal memory base | -1.7 ±.0.89 | -1.8 ±1.3 | -1.8 ±1.1 | 0.68 |
| Long term verbal memory 12m | -1.9 ±0.87 | -2.4 ±1.5 | -2.2 ±1.2 | 0.29 |
| **Rey–Osterrieth** |  |  |  |  |
| Long-term visual memory, base | 6 (4-9) | 2 (2-6) | 5 (2-7) | 0.31 |
| long-term visual memory 12m | 2 (5.5-8) | 2 (2-6) | 3 (2-7) | 0.74 |
| Copy-recall, baseline | 7.1 ±3.5 | 7 ±4.4 | 7 ±3.9 | 0.97 |
| Copy-recall 12m | 6.6 ±2.8 | 5 ±2.4 | 5.8 ±2.7 | 0.08 |

**Supplementary material Table S9:** Cognition among person with amyloid beta <600pg/ml at baseline

Among amyloid-positive participants, women had a greater decline in cognitive performance on the MMSE at 36 months than men, though the difference was not statically significant.
